# Predictive modelling of hypoxic ischaemic encephalopathy risk following perinatal asphyxia

**DOI:** 10.1016/j.heliyon.2021.e07411

**Published:** 2021-06-29

**Authors:** Catherine Mooney, Daragh O'Boyle, Mikael Finder, Boubou Hallberg, Brian H. Walsh, David C. Henshall, Geraldine B. Boylan, Deirdre M. Murray

**Affiliations:** a School of Computer Science, University College Dublin, Dublin, Ireland; b FutureNeuro SFI Research Centre, RCSI University of Medicine and Health Sciences, Dublin, Ireland; c INFANT Research Centre, University College Cork, Cork, Ireland; d Department of Paediatrics and Child Health, University College Cork, Cork, Ireland; e Department of Neonatology, Cork University Maternity Hospital, Cork, Ireland; f Neonatal Department, Karolinska University Hospital, Stockholm, Sweden; g Division of Paediatrics, CLINTEC, Karolinska Institute, Stockholm, Sweden

**Keywords:** Perinatal asphyxia, Hypoxic ischaemic encephalopathy, Clinical risk prediction, Neonatal encephalopathy, Acidosis, Machine learning

## Abstract

Hypoxic Ischemic Encephalopathy (HIE) remains a major cause of neurological disability. Early intervention with therapeutic hypothermia improves outcome, but prediction of HIE is difficult and no single clinical marker is reliable. Machine learning algorithms may allow identification of patterns in clinical data to improve prognostic power. Here we examine the use of a Random Forest machine learning algorithm and five-fold cross-validation to predict the occurrence of HIE in a prospective cohort of infants with perinatal asphyxia. Infants with perinatal asphyxia were recruited at birth and neonatal course was followed for the development of HIE. Clinical variables were recorded for each infant including maternal demographics, delivery details and infant's condition at birth. We found that the strongest predictors of HIE were the infant's condition at birth (as expressed by Apgar score), need for resuscitation, and the first postnatal measures of pH, lactate, and base deficit. Random Forest models combining features including Apgar score, most intensive resuscitation, maternal age and infant birth weight both with and without biochemical markers of pH, lactate, and base deficit resulted in a sensitivity of 56-100% and a specificity of 78-99%. This study presents a dynamic method of rapid classification that has the potential to be easily adapted and implemented in a clinical setting, with and without the availability of blood gas analysis. Our results demonstrate that applying machine learning algorithms to readily available clinical data may support clinicians in the early and accurate identification of infants who will develop HIE. We anticipate our models to be a starting point for the development of a more sophisticated clinical decision support system to help identify which infants will benefit from early therapeutic hypothermia.

## 1 Introduction

HIE occurs in approximately 1-2 per 1,000 term neonates in high-resource settings. It remains a major cause of long term morbidity, with death occurring in 15-20% of cases, and long term adverse neurological outcomes in 25% of those who survive [1], [2], [3]. Currently the only available treatment to mitigate the damage caused by HIE is therapeutic hypothermia, which has been repeatedly confirmed to reduce cerebral injury and improve neurological outcome [4]. Therapeutic hypothermia has been proven to reduce the extent of brain injury if commenced within 6 hours of birth [5]. Due to this limited therapeutic time window and the improved outcomes associated with an early diagnosis, methods of identifying infants who will progress to develop HIE are of great importance. If cooling is commenced even earlier, within 3 hours of birth, outcome is further enhanced [6]. No robust, quantifiable measure of hypoxic brain injury is currently validated to help decide who will benefit most from cooling [7]. Studies examining the implementation of cooling have shown that current assessment methods lack accuracy, with up to 40% of those deemed ineligible by clinicians, actually meeting eligibility criteria. Further complicating this, recent studies indicate that even when accurately applied, current eligibility criteria will miss approximately 20% of infants with significant brain injury [8].

Machine learning can be defined as a computer's ability to learn from data without being explicitly programmed. The use of machine learning in clinical decision making has the potential to affect the lives of millions of patients [9] and transform our understanding of human disease. The use of machine learning in medicine is not new and has been used, for example, in the detection and diagnosis of cancer for decades [10], [11]. More recently, machine learning has been successfully used in medical imaging, including the classification of skin cancer [12] and the detection of diabetic retinopathy [13].

The Random Forest [14] is a machine learning algorithm that operates by ensembling a number of decision trees into a single prediction model. The Random Forest algorithm has been widely used in neurology, for example, Parkinson's disease [15], amyotrophic lateral sclerosis [16], ischemic and hemorrhagic stroke [17], [18] and Alzheimer's disease [19], [20]. Random forests [21] are a popular class of supervised learning models that have demonstrated success across a wide variety of problems. One of the advantages of random forests is their ability to learn high-order, non-linear interactions. Here we use a Random Forest algorithm applied to clinical data that is routinely collected after birth to improve the accuracy of current diagnostic measures for neonatal HIE with the goal of developing a clear method of identifying infants who will benefit from treatment by therapeutic hypothermia within a 6-hour time window.

## 2 Methods

This was a secondary analysis of clinical and biochemical data collected prospectively from infants enrolled into two perinatal asphyxia cohorts with identical recruitment criteria; the first was recruited from May 2009 to May 2011 (BiHiVE study) at Cork University Maternity Hospital and the second from March 2013 to June 2015 (BiHiVE2 study) at both Cork University Maternity Hospital and Karolinska University Hospital, two large maternity services with 8,000 and 4,500 annual deliveries respectively [22]. The BiHiVE2 study was registered on clinicaltrials.gov (NCT02019147) and has been previously reported [23]. Both studies were approved by the Clinical Research Ethics Committee of the Cork Teaching Hospitals, and the BiHiVE2 cohort was also approved by the regional ethical review board in Stockholm, Sweden. Written informed consent was obtained from parents of all study participants. The study team at each centre recorded maternal information, delivery details, the newborn's health at birth, and neonatal course on a study specific internet based database (www.MedSciNet.net/bihive). Source data was cross checked for quality control in 10% of cases across both sites.

Over the combined recruitment periods of 51 months approximately 53,000 deliveries were screened. Inclusion criteria were gestation ≥ 36 weeks and one or more of the following: Cord pH < 7.1, 5-minute Apgar score ≤ 6, the need for intubation or ongoing cardiopulmonary resuscitation at 10 minutes of age. Infants with suspected or confirmed sepsis, or co-existing congenital abnormalities were excluded from the analysis. Infants were then followed throughout their neonatal course and the development of clinical encephalopathy was documented and graded using the modified Sarnat score. Infants with signs of perinatal asphyxia who did not develop HIE were classed as perinatal asphyxia (PA). Maternal, delivery and neonatal data were collected at birth by attending clinical staff and recorded in a study-specific electronic database within the first 72 hours of life. A total of 154 clinical variables were recorded for each birth. Of these, 45 felt to be most relevant and with most complete data were included in our study. Apgar score was assigned by clinical staff attending the delivery. Initial postnatal blood gas values (pH, Base deficit and lactate) were recorded on admission to the neonatal unit following resuscitation. Postnatal blood gas analysis was performed at the discretion of the attending clinician and were either venous or capillary in nature. All infants with clinically suspected HIE in both cohorts received continuous EEG monitoring which was commenced within the first 24 hours of life as previously described [24]. Grade of HIE was assigned using a modified Sarnat score and was later confirmed by analysis of the EEG recordings by a neurophysiologist and expert in neonatal EEG (GB). Infants that had missing values for > 66% of the 45 included clinical variables were excluded from further analysis. A total of 409 infants were included in this study: 208 PA; 68 mild HIE; 44 moderate HIE; and 17 severe HIE. The baseline characteristics of the included infants for each of the 45 clinical variables are listed in Table S1.

## 3 Data preparation

**Figure 1:**
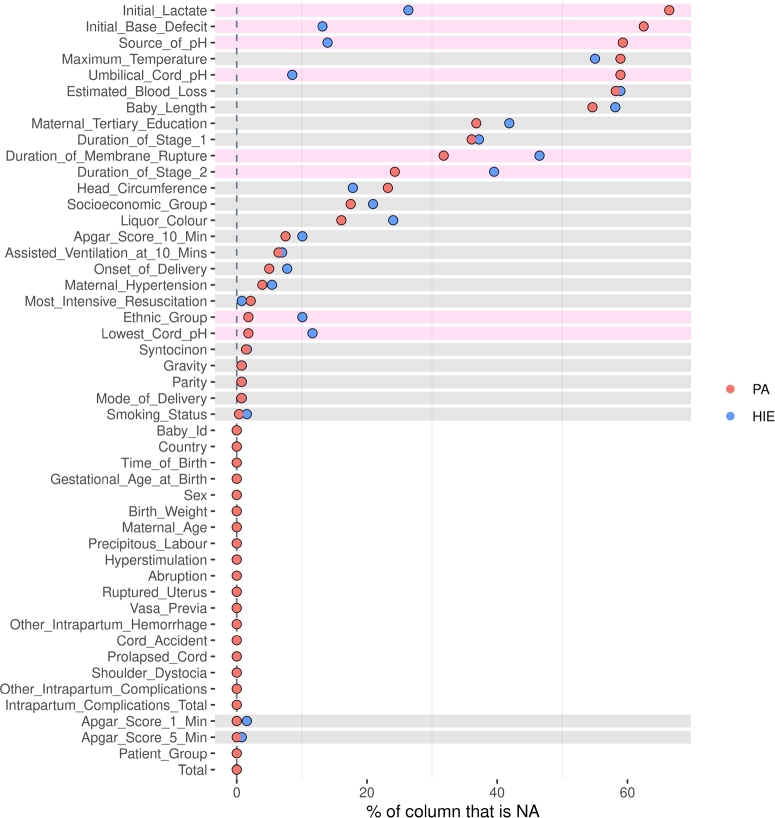
The pattern of “missingness” (NA) between HIE and PA infants for each feature. Pink stripes indicate a different missingness, and grey stripes indicate equal missingness.

Missing values, or “missingness”, in medical datasets is a common problem. There are various causes including subject dropout, missed appointments, unperformed tests or measurements and lost data at data entry [25]. Listwise deletion (omitting observations with missing values), pairwise deletion, and mean substitution (replacing all missing values with average scores) are easy to implement but reduce statistical power and potentially introduce bias [26], [27]. We used the R-package “inspectdf” to compare that pattern of “missingness” between infants with HIE and PA for each feature (Fig. 1). More than 60% of the infants with PA were missing biochemical markers of pH, lactate and base deficit. As this was performed at clinician discretion they possibly had a milder clinical presentation, affecting predictive ability. Therefore, we developed two models. The first, where the features pH, lactate and base deficit were removed from the dataset (Model 1), and the second where infants without pH, lactate and base deficit were removed from the dataset (Model 2). Furthermore, “Maximum Temperature” “Estimated Blood Loss” and “Baby length” were removed from both models as they had > 50% missingness.

**Figure 2:**
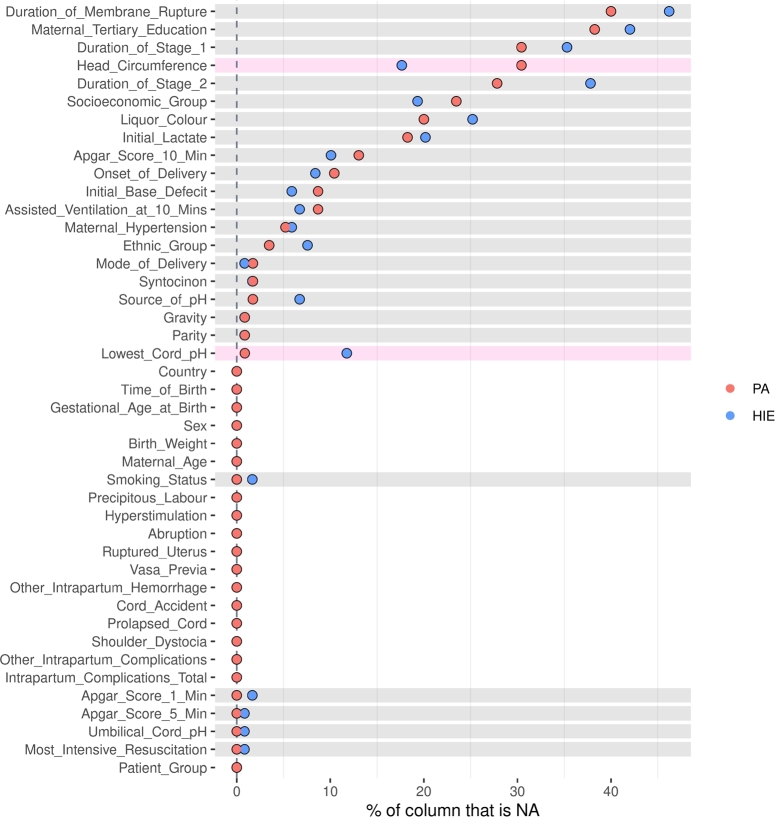
The pattern of “missingness” (NA) between HIE and PA infants for each feature in the Model 2 data after the removal of 175 infants that were missing pH, lactate and base deficit. Pink stripes indicate a different missingness, and grey stripes indicate equal missingness.

**Figure 3:**
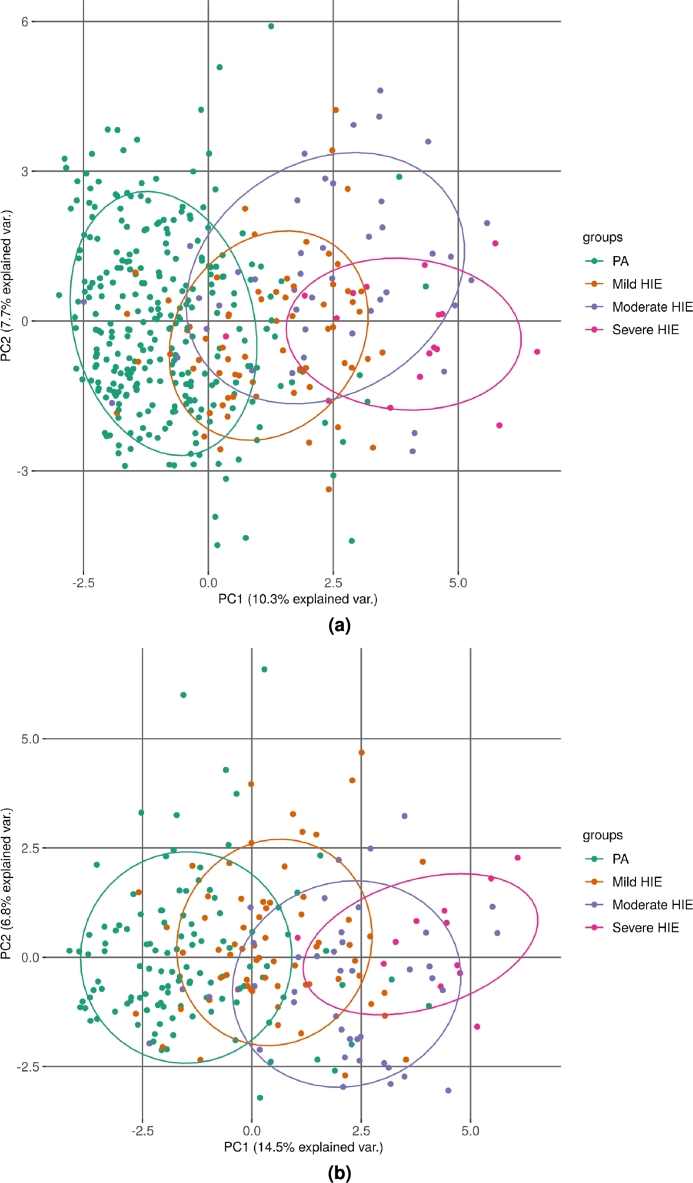
Principal component analysis (a) after removal of biochemical markers of pH, lactate and base deficit and lowest cord pH followed by imputations of missing values (Model 1) and (b) after removal of infants with missing pH, lactate and base deficit (Model 2). PA: perinatal asphyxia without encephalopathy, HIE: hypoxic ischaemic encephalopathy.

We additionally removed the lowest cord pH from Model 1, even though the missingness was < 20%. The logic for this was that Model 1 could then be developed for use in a low resource setting as no blood tests would be required whereas Model 2 is intended for use in a setting where pre- and postnatal blood sampling is readily available. After further inspection of Model 2 data after the removal of 175 infants that were missing pH, lactate and base deficit (Fig. 2) we removed the duration of ruptured membranes, maternal tertiary education, duration of labour stage 1 and 2, and baby's head circumference as they had > 25% missingness. We used the R-package “mice” [28] with default parameters to impute the remaining missing data for both datasets. The default imputation method depends on the measurement level of the target column and in this case was predictive mean matching. Fig. 3 shows the principal component analysis for Model 1 and 2 after imputation of missing values.

The datasets were split into a training and independent test set (70%/30% respectively), and then the training set was further split into a training and validation set (70%/30% respectively) (Table 1). A random forest was trained in five-fold cross-validation. The hyper-parameters of the Random Forest were selected using a grid search on the training set in five-fold cross-validation. The highest accuracy for Model 1 was achieved with an “mtry” of 5, a minimum node size of 2 and 200 trees, using Gini impurity as the “splitrule”. The highest accuracy for Model 2 was achieved with an “mtry” of 2, a minimum node size of 2 and 200 trees, using Gini impurity as the “splitrule”.

**Table 1:** Table showing the number of infants in each category in the training, validation and independent test sets for Model 1 (biochemical markers of pH, lactate, base deficit and lowest cord pH removed) and Model 2 (infants with missing pH, lactate and base deficit removed). PA = perinatal asphyxia without encephalopathy, HIE = hypoxic ischaemic encephalopathy (all grades), Mild = mild HIE, Mod = moderate HIE, Severe = severe HIE.

	Model 1					Model 2				
	PA	HIE	Mild	Mod	Severe	PA	HIE	Mild	Mod	Severe
Training	141	61	30	22	9	57	59	32	20	7
Validation	59	26	14	9	3	22	26	15	10	1
Independent	80	42	24	13	5	36	34	18	11	5
Total	280	129	68	44	17	115	119	65	41	13

### 3.1 Feature selection

**Figure S1:**
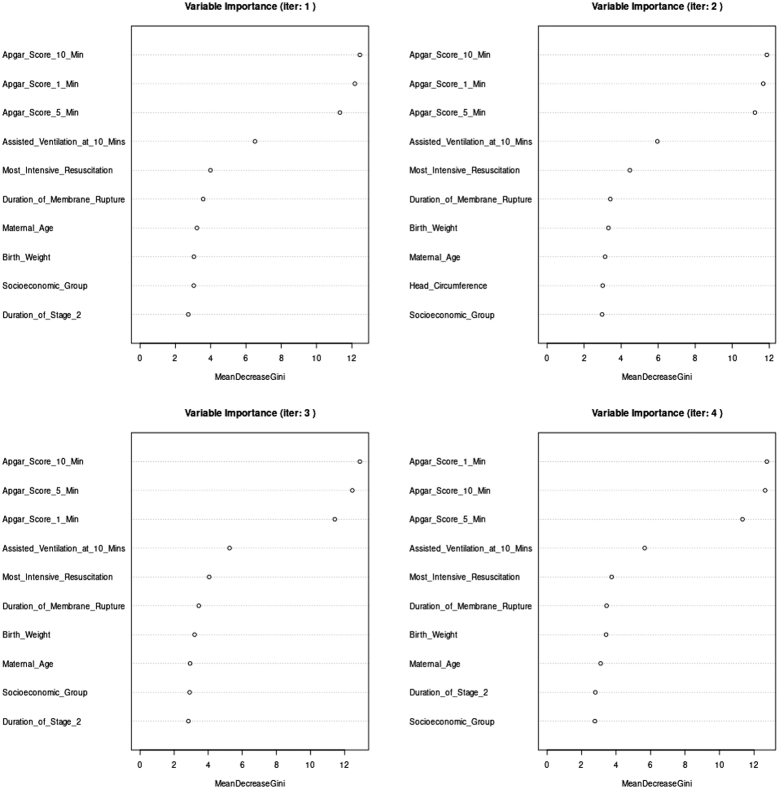
Variable importance plots for Model 1 (biochemical markers of pH, lactate and base deficit and lowest cord pH removed).

**Figure S2:**
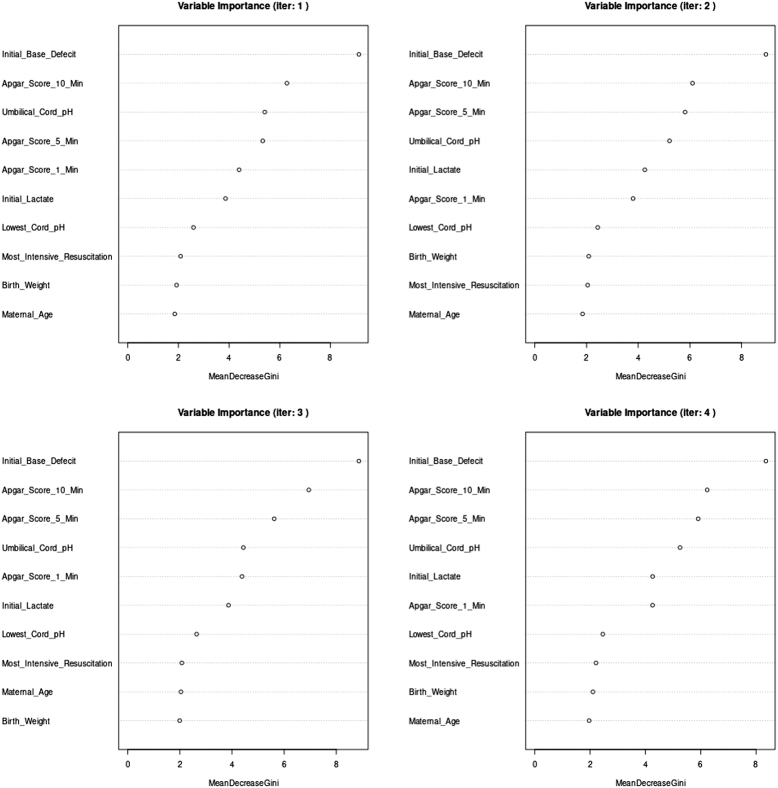
Variable importance plots for Model 2 (infants with missing pH, lactate and base deficit removed).

For each machine learning task, there exists a unique subset of features that are most important for high classification accuracy. Identifying the optimal combination of features to train a classifier is essential as it improves system accuracy and computational efficiency. We used iterative random forests (iRF) [14] for feature selection as it can identify important feature interactions. The iRF weights features according to feature importance, growing more relevant trees to uncover complex interactions. The stability score describes the fraction of times an interaction occurs, with stable interactions having scores greater than 0.5. A higher stability score means it is less likely that random chance alone caused identification of the interaction [29]. Table 2 shows the interactions recovered by iRF with stability score > 0.5 for both Model 1 and Model 2 on the validation set. Feature selection was performed based on the iRF interactions and the variable importance plots as shown in Figures S1 and S2. Variable importance is measured by the mean decrease in Gini impurity, a higher mean decrease in Gini impurity indicates a higher variable importance. The features selected for both models are shown in Table 3.

**Table 2:** Interactions recovered by iRF with stability score > 0.5. Model 1 (biochemical markers of pH, lactate and base deficit and lowest cord pH removed) and Model 2 (infants with missing pH, lactate and base deficit removed).

Model 1		Model 2	
1-min Apgar + 10-min Apgar	1	5-min Apgar + 10-min Apgar	1
5-min Apgar + 10-min Apgar	1	10-min Apgar + Initial base deficit	1
1-min Apgar + 5-min Apgar	0.97	5-min Apgar + Initial base deficit	0.97
Duration of membrane rupture, h + 10-min Apgar	0.90	10-min Apgar + Lowest cord pH	0.80
Birthweight, g + 1-min Apgar	0.90	1-min Apgar + Initial base deficit	0.77
Socio-economic group + 10-min Apgar	0.87	Umbilical cord pH + Initial base deficit	0.73
Birthweight, g + 10-min Apgar	0.87	1-min Apgar + 10-min Apgar	0.70
Socio-economic group + 5-min Apgar	0.83	10-min Apgar + Umbilical cord pH	0.70
Socio-economic group + 1-min Apgar	0.80	10-min Apgar + Initial lactate	0.67
Duration of membrane rupture, h + 1-min Apgar	0.80	Maternal age + Initial base deficit	0.67
Duration of stage 2 + 10-min Apgar	0.73	5-min Apgar + Lowest cord pH	0.60
Duration of membrane rupture, h + 5-min Apgar	0.70	5-min Apgar + Umbilical cord pH	0.60
10-min Apgar + Head circumference	0.70	5-min Apgar + Initial lactate	0.57
Birthweight, g + 5-min Apgar	0.70	Lowest cord pH + Initial base deficit	0.57
Maternal age + 1-min Apgar	0.70	Birthweight, g + Initial base deficit	0.53
Maternal age + 10-min Apgar	0.70		
1-min Apgar + Head circumference	0.63		
Maternal age + 5-min Apgar	0.63		
Duration of stage 2 + 1-min Apgar	0.60		
5-min Apgar + Head circumference	0.57		
Time of birth, 24 h + 10-min Apgar	0.53

**Table 3:** Table showing the features selected for each model. Features were selected based on the iRF interactions (Table 2) and the variable importance plots (Figures S1 and S2). ^⁎^ identifies feature appearing in the variable importance plots only and ^⁎⁎^ identifies feature selected based on the iRF interactions only.

Model 1	Model 2
Apgar Score 1	Apgar Score 1
Apgar Score 5	Apgar Score 5
Apgar Score 10	Apgar Score 10
Most Intensive Resuscitation^⁎^	Most Intensive Resuscitation^⁎^
Maternal Age	Maternal Age
Birth Weight	Birth Weight
Time of Birth^⁎⁎^	First Postnatal Base Deficit
Assisted Ventilation at 10 mins^⁎^	First Postnatal Lactate
Head Circumference^⁎⁎^	First Postnatal pH
Duration of Second Stage	Lowest Cord pH
Duration Membrane Rupture	
Socioeconomic Group	

### 3.2 Model evaluation and validation

To evaluate the performance of our models we measured accuracy, specificity, sensitivity, precision, the false positive rate (FRP) and Matthews correlation coefficient (MCC) on the independent test set as follows:

$$Accuracy=\frac{TP+TN}{TP+TN+FP+FN}\;Specificity=\frac{TN}{TN+FP}\;Sensitivity=\frac{TP}{TP+FN}\;Precision=\frac{TP}{TP+FP}\;FPR=\frac{FP}{FP+TN}\;MCC=\frac{TP\times TN-FP\times FN}{\sqrt{\left(TP+FP)(TP+FN)(TN+FP)(TN+FN\right)}}$$

where:

• True positives (TP): the number of infants predicted as HIE that are observed in the HIE class.
• False positives (FP): the number of infants predicted as HIE that are not observed in the HIE class.
• True negatives (TN): the number of infants predicted as PA that are observed in the PA class.
• False negatives (FN): the number of infants predicted as PA that are not observed in the PA class.

## 4 Results

Of the 53,000 screened deliveries, 129 infants with HIE were recruited; giving a rate of HIE of 2.4 per 1,000 live births (similar to expected in the high income settings studied). Infants with neonatal stroke, sepsis during NICU admission or other rare conditions (e.g. metabolic disorders, congenital cytomegalovirus (CMV), hypoglycemic brain damage or seizures of unknown aetiology) were excluded from the analysis. We retained 409 single birth infants (237 males and 172 females) with a confirmed diagnosis of PA (n = 280), mild HIE (n = 68), moderate HIE (n = 44) or severe HIE (n = 17) from two centres, Cork University Maternity Hospital (n = 260) and Karolinska University Hospital (n = 149), of primarily Caucasian descent (83%).

As more than 60% of the PA infants were missing biochemical markers of pH, lactate and base deficit we developed two models. The first model (Model 1) does not include these biochemical markers as features whereas the second model does not include infants that were missing values for these biochemical markers (Model 2). Finally, 409 and 234 infants were included in the Model 1 and Model 2 datasets respectively. Principal component analysis shows distinct discrimination between PA and severe HIE infants by the first component (28.3% of total variance) (Fig. 3). However the mild and moderate HIE infants are largely overlapping in both models, in concordance with the clinical picture, where these grades are difficult to differentiate.

The features used to train each model were selected based on the iRF interactions (Table 2) and the variable importance plots (Figures S1 and S2) on the validation sets. Apgar Score at 1, 5 and 10 minutes, the most intensive resuscitation required at birth (i.e. none, facial oxygen, BMV/IPPV, CPAP/PEEP, intubation, CPR, CPR and adrenaline), maternal age and birth weight were included in both models (Table 3). The time of birth, head circumference, duration of the second stage of labour, the duration of membrane rupture, socioeconomic group and if assisted ventilation was required at 10 minutes were additionally included in Model 1. First postnatal base deficit, first postnatal lactate, first postnatal pH and lowest cord pH were additionally include in Model 2 (Table 3).

**Figure 4:**
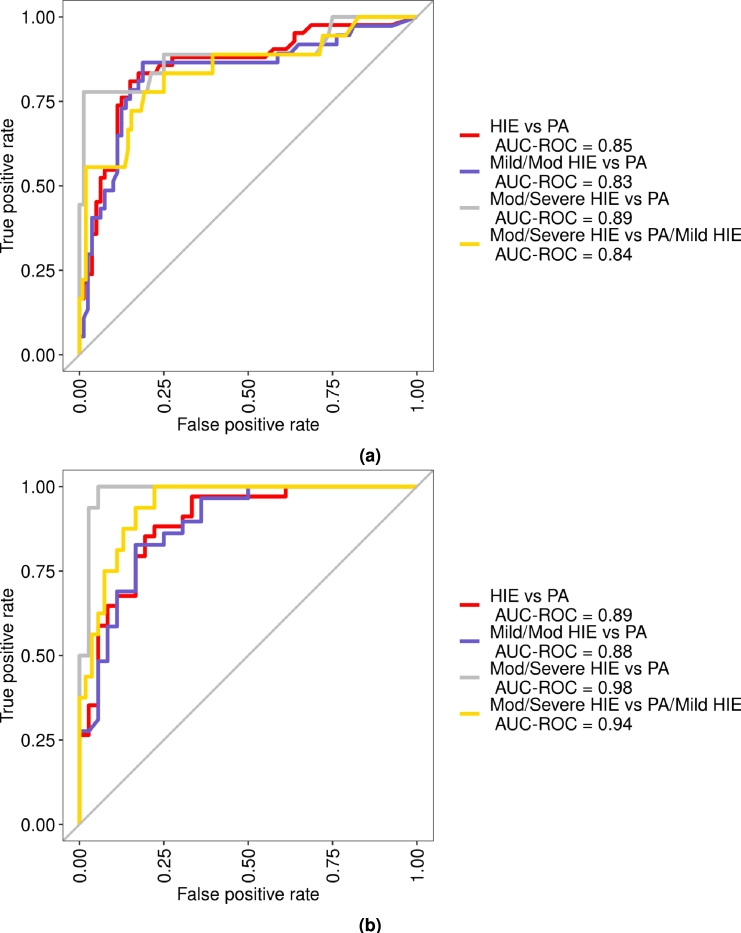
ROC plots (a) after removal of biochemical markers of pH, lactate and base deficit and lowest cord pH followed by imputations of missing values (Model 1) and (b) after removal of infants with missing pH, lactate and base deficit (Model 2). PA: perinatal asphyxia without encephalopathy, HIE: hypoxic ischaemic encephalopathy.

Tables 4 and S2 show the evaluation of Model 1 (no biochemical markers) and Tables 5 and S3 show the evaluation of Model 2 (including biochemical markers) on the independent test set. In both cases we trained independent random forest to discriminate between: PA versus all grades of HIE; PA versus mild and moderate HIE; PA versus moderate and severe HIE; and PA and mild HIE versus moderate and severe HIE. Model 1 (without biochemical markers) performed well at discriminating between PA and HIE (accuracy 0.83; specificity 0.89, sensitivity 0.71). Model 1 was also able to identify those children most likely to develop moderate/severe HIE (accuracy 0.94, specificity 0.99, sensitivity 0.72). Similarly Model 2 (with biochemical markers) displayed a good ability to discriminate between PA and all HIE grades (accuracy 0.81, specificity 0.78, sensitivity 0.85) and improved ability to predict those with moderate/severe HIE (accuracy 0.94, specificity 0.92, sensitivity 1) indicating ability to predict the severity of the HIE grade. ROC plots for Model 1 and 2 are shown in Fig. 4.

**Table 4:** Table showing the evaluation of Model 1 (no biochemical markers) on the independent test set. PA = perinatal asphyxia without encephalopathy, HIE = hypoxic ischaemic encephalopathy, Mild = mild HIE, Mod = moderate HIE, Severe = severe HIE, FPR = false positive rate, MCC = Matthews correlation coefficient.

	PA vs HIE (all grades)	PA vs Mild/Mod	PA vs Mod/Severe	PA/Mild vs Mod/Severe
Accuracy	0.83	0.80	0.94	0.91
Specificity	0.89	0.88	0.99	0.97
Sensitivity	0.71	0.65	0.72	0.56
Precision	0.77	0.71	0.93	0.77
FPR	0.11	0.13	0.01	0.03
MCC	0.61	0.54	0.79	0.61

**Table 5:** Table showing the evaluation of Model 2 (including biochemical markers) on the independent test set. PA = perinatal asphyxia without encephalopathy, HIE = hypoxic ischaemic encephalopathy, Mild = mild HIE, Mod = moderate HIE, Severe = severe HIE, FPR = false positive rate, MCC = Matthews correlation coefficient.

	PA Vs HIE (all grades)	PA Vs Mild/Mod	PA Vs Mod/Severe	PA/Mild Vs Mod/Severe
Accuracy	0.81	0.82	0.94	0.86
Specificity	0.78	0.81	0.92	0.93
Sensitivity	0.85	0.83	1.00	0.63
Precision	0.78	0.77	0.84	0.71
FPR	0.22	0.19	0.08	0.07
MCC	0.63	0.63	0.88	0.58

## 5 Discussion

Applying machine learning algorithms to readily available clinical data may support the early and accurate identification of infants who will develop HIE. Through the use of iRF and variable importance plots, we have identified a subset of features to predict HIE with the goal of creating a parsimonious model. The number of features has been reduced from 154 potential clinical predictors to 10 or 12. The strongest predictors were the Apgar Score at 1, 5 and 10 minutes, the need for resuscitation, maternal age, and infant birth weight, with the addition of the first postnatal measures of lactate, pH, and base deficit, if available. This will allow for faster and easier classification of patients when using this method. Although these markers have been recorded and used to determine HIE risk prediction for many years, they have often been used as dichotomous variables (i.e. pH < 7.1, Base deficit > 16). Their strength, for prediction, however lies in their changes across the spectrum of acidosis, as continuous variables. Being able to calculate risk using these indices as continuous variables greatly improves the strength and reliability of the model. The implementation of our models as decision support will be facilitated by the move of many centres to universal electronic health records. In-built algorithms could help to alert clinicians regarding the risk of HIE, and more importantly the likelihood of moderate/severe HIE, in order to guide the need for further monitoring, and initiation of therapeutic hypothermia. Although clinical guidelines recommend cooling therapy only for those with moderate or severe HIE, there has been a very strong therapeutic drift, with many centres in the US and UK cooling all grades of encephalopathy [30]. Thus both prediction of HIE and prediction of grade are equally important.

We have developed two models. Model 1 does not include biochemical data and could be developed for use in a low resource setting as no blood tests would be required whereas Model 2 is intended for use in a setting where pre- and postnatal blood sampling is readily available. Apgar Score at 1, 5 and 10 minutes, the most intensive resuscitation required at birth, maternal age and birth weight were included in both models. When biochemical analysis of acid base status was available, this added significantly to the model (Model 2). However, when biochemical data was not available, clinical markers (assisted ventilation at 10 mins, infant head circumference, duration of second stage of labour, duration of membrane rupture and maternal SE group) can be used, to increase the predictive value. The variables of most importance in the model were those reflecting the infant's condition at birth (Apgar scores and most intensive resuscitation). Maternal age, birth weight and infant head size give an indication of the risk of obstructed or prolonged labour, whilst duration of second stage is a measure of labour progression. Finally the acid base derangement measurable on the first postnatal blood gas gives an indication of the effectiveness of, and infant's response to, early resuscitation.

There have been numerous applications of machine learning for the improvement of the treatment of neonatal HIE to date, with each providing unique benefits to the clinician. Machine learning has been utilised for the automation of EEG signals in order to aid in the detection of seizures in HIE neonates [31], [32]. This improves the ability to diagnose and grade severity in areas where specialists are not available for EEG readings. Machine learning has also been applied to aid in the interpretation of MRI readings in order to predict mortality and long-term neurological disability [33], [34], [35]. Despite benefits in determining long term neurological outcomes these methods are not accessible without the use of specialised equipment (EEG, MRI) and will not aid in identifying those in need of treatment from therapeutic hypothermia within the 6-hour treatment window. Machine learning methods have also been applied to readily available clinical data in order to assist clinicians treating neonates with HIE. Slattery et al. [36] used clinical data collected over the first seven days of life and maternal data on pregnancy and birth to predict mortality risk with a median accuracy of 72%. O'Boyle et al. [37] combined routinely collected clinical data (Apgar scores, blood gases) with umbilical cord metabolites alanine and lactic acid to distinguish between those who will develop HIE from those with PA and healthy controls with an accuracy of 97.3%. However, this study is the only machine learning based HIE study to demonstrate the ability of routinely collected clinical data alone for the prediction of HIE grade. It provides the advantage of not requiring specialist equipment and training for data collection and interpretation and therefore has the potential to be used in a wide variety of clinical settings while still retaining high predictive power. It is also applicable from as early as 1 hour from birth, with or without the availability of blood gases, resulting it the potential of the model to guide treatment at a time where intervention may have the highest impact.

Study Limitations: This study took place in an inborn population of infants, in tertiary centres in two countries with advanced obstetric care and newborn care, where all neonatal staff have been trained in newborn resuscitation. Thus its utility will have to be examined in other settings, in regional hospitals and low or middle income countries, where the rates of severe HIE may be higher, and staff may not be specifically trained in newborn resuscitation.

We must also consider that there may be potential bias in the dataset for Model 2 as not all infants with PA had postnatal blood gas analysis performed. Those who had postnatal bloods drawn are likely to have had a more severe clinical presentation and thus be more similar to infants with HIE. The cohort tested by Model 2 therefore, had a higher pretest probability prior to analysis. This may have affected Model 2's ability to differentiate between PA and HIE. However, our data reflect what occurs in the clinical setting. Clinicians currently use their experience to decide on the need for blood gas analysis. Perhaps if this was part of an available clinical decision tool, it may become standard of care for all infants with evidence of perinatal compromise.

This study presents a dynamic method of rapid classification that has the potential to be easily adapted and implemented in a clinical setting, with and without the availability of blood gas analysis. The main advantage of this technique is that it can be used at one hour after birth when post resuscitation gases are available. This will help determine those who will benefit from treatment with therapeutic hypothermia within the 6 hour time window. In fact, a rapid risk identifier could enable the initiation of therapeutic hypothermia within 3 hours after birth. Early cooling (< 3 hours) may lead to improved outcomes as it has previously been shown to be even more effective [6]. To para-phrase the authors “Time is brain”. A quantifiable risk calculator would be particularly useful for regional hospitals to aid communication with tertiary hospital and ensure rapid transfer for neuroprotective interventions and neuro-intensive care.

## 6 Conclusion

We have shown that, through the use of readily available clinical data with machine learning classification techniques, we can improve our ability to predict the development of HIE following perinatal asphyxia. These models can accurately identified infants who will develop HIE from those who will recover quickly and have a normal neonatal outcome. Readily available clinical markers, even in settings where blood gas analysis is not possible, can be used to predict the risk of HIE and the risk of moderate-severe grade HIE. This could be incorporated into bedside decision support tools to ensure targeted and rapid initiation of therapeutic hypothermia in those infants to whom it would benefit most.

## Declarations

### Author contribution statement

Catherine Mooney: Conceived and designed the experiments; Performed the experiments; Analyzed and interpreted the data; Wrote the paper.

Daragh O'Boyle: Performed the experiments; Analyzed and interpreted the data; Wrote the paper.

Mikael Finder, Boubou Hallberg, Brian H. Walsh: Contributed reagents, materials, analysis tools or data.

David C. Henshall: Conceived and designed the experiments.

Geraldine B. Boylan: Conceived and designed the experiments; Contributed reagents, materials, analysis tools or data.

Deirdre M. Murray: Conceived and designed the experiments; Performed the experiments; Analyzed and interpreted the data; Contributed reagents, materials, analysis tools or data; Wrote the paper.

### Funding statement

This work was supported by 10.13039/501100001602Science Foundation Ireland (SFI) under Grant Numbers SFI/12/RC/2272 (to D.M.M. and G.B.B.), SFI/16/RC/3948 and SFI/14/ADV/RC2721 (to D.C.H. and C.M.) and co-funded under the 10.13039/501100008530European Regional Development Fund and by FutureNeuro and INFANT industry partners. The BiHiVE 2 study was funded by the HRB Clinician Scientist Award CSA 2012/40 (to D.M.M.).

### Data availability statement

The data that has been used is confidential.

### Declaration of interests statement

The authors declare no conflict of interest.

### Additional information

Supplementary content related to this article has been published online at https://doi.org/10.1016/j.heliyon.2021.e07411.

No additional information is available for this paper.

## Supplementary material

The following Supplementary material is associated with this article:

Demographics_full.pdf Clinical Demographics. The baseline characteristics of the included infants for each of the 45 clinical variables. PA: perinatal asphyxia without encephalopathy, HIE: hypoxic ischaemic encephalopathy, Mild: mild HIE, Moderate: moderate HIE, Severe: severe HIE, SD: standard deviation; ARM: artificial rupture of membrane; Emer CS: emergency Caesarean section; LSCS: lower segment Caesarean section; ABG: arterial blood gases; VBG: venous blood gases; CBG: capillary blood gas; BMV: bag valve mask; IPPV: intermittent positive pressure ventilation; CPR: cardio-pulmonary resuscitation; CPAP: continuous positive airway pressure; PEEP: positive end expiratory pressure. Demographics_full.pdf

Pages from HLY7411_source.pdf Table showing the evaluation of Model 1 (no biochemical markers) on the independent test set. PA = perinatal asphyxia without encephalopathy, HIE = hypoxic ischaemic encephalopathy, Mild = mild HIE, Mod = moderate HIE, Severe = severe HIE, TP = true positive, FP = false positive, TN = true negative, FN = false negative. Pages from HLY7411_source.pdf

Pages from HLY7411_source.pdf Table showing the evaluation of Model 2 (including biochemical markers) on the independent test set. PA = perinatal asphyxia without encephalopathy, HIE = hypoxic ischaemic encephalopathy, Mild = mild HIE, Mod = moderate HIE, Severe = severe HIE, TP = true positive, FP = false positive, TN = true negative, FN = false negative. Pages from HLY7411_source.pdf
