# Supplementary material for: Predictive modelling of hypoxic ischaemic encephalopathy risk following perinatal asphyxia
Source: Heliyon. 2021 Jun 29;7(7):e07411. doi: 10.1016/j.heliyon.2021.e07411 (PMC8261660; doi:10.1016/j.heliyon.2021.e07411)
Supplement: Demographics_full.pdf — Clinical Demographics. The baseline characteristics of the included infants for each of the 45 clinical variables. PA: perinatal asphyxia without encephalopathy, HIE: hypoxic ischaemic encephalopathy, Mild: mild HIE, Moderate: moderate HIE, Severe: severe HIE, SD: standard deviation; ARM: artificial rupture of membrane; Emer CS: emergency Caesarean section; LSCS: lower segment Caesarean section; ABG: arterial blood gases; VBG: venous blood gases; CBG: capillary blood gas; BMV: bag valve mask; IPPV: intermittent positive pressure ventilation; CPR: cardio-pulmonary resuscitation; CPAP: continuous positive airway pressure; PEEP: positive end expiratory pressure. [file mmc1.pdf]

	PA (n=280)	Mild (n=68)	Moderate (n=44)	Severe (n=17)	Overall (n=409)
**Country**					
Ireland	169 (60.4%)	57 (83.8%)	20 (45.5%)	14 (82.4%)	260 (63.6%)
Sweden	111 (39.6%)	11 (16.2%)	24 (54.5%)	3 (17.6%)	149 (36.4%)
**Time of birth**					
Mean (SD)	10.7 (7.48)	11.8 (8.06)	11.1 (8.01)	11.2 (7.57)	10.9 (7.63)
Median [Min, Max]	10.0 [0.00, 23.0]	10.5 [0.00, 23.0]	11.0 [0.00, 23.0]	13.0 [1.00, 23.0]	10.0 [0.00, 23.0]
**Gestational age at birth, d**					
Mean (SD)	281 (8.95)	280 (9.01)	282 (9.59)	281 (7.16)	281 (8.94)
Median [Min, Max]	283 [252, 295]	282 [252, 292]	283 [252, 297]	280 [266, 291]	282 [252, 297]
**Sex**					
Male	155 (55.4%)	43 (63.2%)	28 (63.6%)	11 (64.7%)	237 (57.9%)
Female	125 (44.6%)	25 (36.8%)	16 (36.4%)	6 (35.3%)	172 (42.1%)
**Birth weight, g**					
Mean (SD)	3600 (525)	3560 (499)	3670 (553)	3580 (598)	3600 (526)
Median [Min, Max]	3590 [2330, 5640]	3500 [2570, 4710]	3560 [2100, 4750]	3430 [2880, 5190]	3550 [2100, 5640]
**Maternal age**					
Mean (SD)	31.5 (5.44)	31.1 (5.19)	29.8 (5.06)	31.9 (5.45)	31.2 (5.37)
Median [Min, Max]	32.0 [18.0, 46.0]	31.0 [19.0, 45.0]	30.5 [20.0, 38.0]	32.0 [22.0, 44.0]	31.0 [18.0, 46.0]
**Gravity**					
Mean (SD)	2.01 (1.43)	1.96 (1.11)	1.68 (1.03)	2.18 (1.51)	1.98 (1.35)
Median [Min, Max]	2.00 [1.00, 10.0]	2.00 [1.00, 5.00]	1.00 [1.00, 4.00]	2.00 [1.00, 6.00]	2.00 [1.00, 10.0]
Missing	2 (0.7%)	1 (1.5%)	0 (0%)	0 (0%)	3 (0.7%)
**Parity**					
Mean (SD)	1.58 (1.00)	1.58 (0.924)	1.34 (0.805)	1.53 (0.717)	1.55 (0.959)
Median [Min, Max]	1.00 [0.00, 8.00]	1.00 [0.00, 4.00]	1.00 [0.00, 4.00]	1.00 [1.00, 3.00]	1.00 [0.00, 8.00]
Missing	2 (0.7%)	1 (1.5%)	0 (0%)	0 (0%)	3 (0.7%)
**Ethnic group**					
Caucasian	233 (83.2%)	60 (88.2%)	34 (77.3%)	14 (82.4%)	341 (83.4%)
African	18 (6.4%)	3 (4.4%)	1 (2.3%)	1 (5.9%)	23 (5.6%)
Asian	24 (8.6%)	3 (4.4%)	0 (0%)	0 (0%)	27 (6.6%)
Missing	5 (1.8%)	2 (2.9%)	9 (20.5%)	2 (11.8%)	18 (4.4%)
**Smoking status**					
Non smoker	238 (85.0%)	56 (82.4%)	37 (84.1%)	11 (64.7%)	342 (83.6%)
Smoked prior to pregnancy	25 (8.9%)	8 (11.8%)	4 (9.1%)	2 (11.8%)	39 (9.5%)
Smoked during pregnancy	16 (5.7%)	4 (5.9%)	2 (4.5%)	3 (17.6%)	25 (6.1%)
Missing	1 (0.4%)	0 (0%)	1 (2.3%)	1 (5.9%)	3 (0.7%)
**Maternal hypertension**					
No hypertension	250 (89.3%)	61 (89.7%)	40 (90.9%)	15 (88.2%)	366 (89.5%)
Essential hypertension	1 (0.4%)	0 (0%)	1 (2.3%)	0 (0%)	2 (0.5%)
Pre-eclampsia	18 (6.4%)	4 (5.9%)	1 (2.3%)	0 (0%)	23 (5.6%)
Missing	11 (3.9%)	3 (4.4%)	2 (4.5%)	2 (11.8%)	18 (4.4%)
**Maternal tertiary education**					
No	79 (28.2%)	13 (19.1%)	7 (15.9%)	3 (17.6%)	102 (24.9%)
Yes	98 (35.0%)	30 (44.1%)	12 (27.3%)	10 (58.8%)	150 (36.7%)
Missing	103 (36.8%)	25 (36.8%)	25 (56.8%)	4 (23.5%)	157 (38.4%)
**Socio-economic group**					
Higher managerial	63 (22.5%)	22 (32.4%)	6 (13.6%)	4 (23.5%)	95 (23.2%)
Intermediate occupations	30 (10.7%)	11 (16.2%)	10 (22.7%)	4 (23.5%)	55 (13.4%)
Small employers	13 (4.6%)	3 (4.4%)	1 (2.3%)	1 (5.9%)	18 (4.4%)
Lower supervisory	26 (9.3%)	3 (4.4%)	3 (6.8%)	4 (23.5%)	36 (8.8%)
Semi-routine/routine	57 (20.4%)	10 (14.7%)	6 (13.6%)	1 (5.9%)	74 (18.1%)
Never worked/long-term unemployed/students	42 (15.0%)	8 (11.8%)	4 (9.1%)	1 (5.9%)	55 (13.4%)
Missing	49 (17.5%)	11 (16.2%)	14 (31.8%)	2 (11.8%)	76 (18.6%)
**Onset of delivery**					
Spontaneous	156 (55.7%)	34 (50.0%)	31 (70.5%)	9 (52.9%)	230 (56.2%)
ARM	26 (9.3%)	5 (7.4%)	2 (4.5%)	3 (17.6%)	36 (8.8%)
Pharmacological Induction	76 (27.1%)	17 (25.0%)	7 (15.9%)	1 (5.9%)	101 (24.7%)
Prelabour Caesarean	7 (2.5%)	3 (4.4%)	3 (6.8%)	1 (5.9%)	14 (3.4%)
Mechanical Induction/Bard Catheter	1 (0.4%)	2 (2.9%)	0 (0%)	1 (5.9%)	4 (1.0%)
Missing	14 (5.0%)	7 (10.3%)	1 (2.3%)	2 (11.8%)	24 (5.9%)
**Duration of membrane rupture, h**					
Mean (SD)	12.5 (18.4)	15.6 (18.4)	15.7 (15.2)	12.3 (11.0)	13.2 (17.9)

	PA (n=280)	Mild (n=68)	Moderate (n=44)	Severe (n=17)	Overall (n=409)
Median [Min, Max]	8.00 [1.00, 192]	8.00 [1.00, 70.0]	11.0 [1.00, 58.0]	11.5 [1.00, 27.0]	8.00 [1.00, 192]
Missing	89 (31.8%)	32 (47.1%)	17 (38.6%)	11 (64.7%)	149 (36.4%)
**Duration of stage 1**					
Mean (SD)	6.26 (4.13)	6.21 (4.09)	7.98 (4.56)	6.44 (5.00)	6.42 (4.20)
Median [Min, Max]	6.00 [1.00, 30.0]	6.00 [0.700, 20.0]	8.00 [0.00, 16.0]	6.00 [0.00, 14.0]	6.00 [0.00, 30.0]
Missing	101 (36.1%)	21 (30.9%)	19 (43.2%)	8 (47.1%)	149 (36.4%)
**Durationof stage 2**					
Mean (SD)	82.7 (69.4)	59.6 (51.8)	85.8 (80.3)	83.6 (65.3)	79.3 (68.0)
Median [Min, Max]	64.0 [2.00, 300]	50.0 [0.00, 178]	52.5 [0.00, 300]	57.5 [7.00, 195]	58.0 [0.00, 300]
Missing	68 (24.3%)	22 (32.4%)	20 (45.5%)	9 (52.9%)	119 (29.1%)
**Maximum Temperature**					
Mean (SD)	37.1 (0.584)	37.2 (0.495)	37.7 (0.956)	36.9 (0.361)	37.2 (0.640)
Median [Min, Max]	37.1 [35.9, 39.2]	37.1 [36.3, 38.4]	37.6 [36.4, 39.7]	36.8 [36.6, 37.3]	37.1 [35.9, 39.7]
Missing	165 (58.9%)	33 (48.5%)	24 (54.5%)	14 (82.4%)	236 (57.7%)
**Mode of delivery**					
Unassisted vaginal	88 (31.4%)	18 (26.5%)	5 (11.4%)	2 (11.8%)	113 (27.6%)
Assisted (Forceps)	29 (10.4%)	10 (14.7%)	1 (2.3%)	4 (23.5%)	44 (10.8%)
Assisted (Ventouse)	114 (40.7%)	22 (32.4%)	23 (52.3%)	2 (11.8%)	161 (39.4%)
Failed assisted and Emer CS	4 (1.4%)	3 (4.4%)	0 (0%)	0 (0%)	7 (1.7%)
Prelabour LSCS	8 (2.9%)	1 (1.5%)	3 (6.8%)	1 (5.9%)	13 (3.2%)
Emer CS in labour	35 (12.5%)	14 (20.6%)	11 (25.0%)	8 (47.1%)	68 (16.6%)
Missing	2 (0.7%)	0 (0%)	1 (2.3%)	0 (0%)	3 (0.7%)
**Estimated Blood Loss**					
Mean (SD)	658 (445)	488 (251)	638 (376)	545 (317)	632 (413)
Median [Min, Max]	500 [120, 2190]	425 [150, 1100]	450 [200, 1700]	500 [175, 1050]	495 [120, 2190]
Missing	163 (58.2%)	48 (70.6%)	16 (36.4%)	12 (70.6%)	239 (58.4%)
**Syntocinon**					
No	134 (47.9%)	42 (61.8%)	18 (40.9%)	12 (70.6%)	206 (50.4%)
Yes	142 (50.7%)	26 (38.2%)	25 (56.8%)	4 (23.5%)	197 (48.2%)
Missing	4 (1.4%)	0 (0%)	1 (2.3%)	1 (5.9%)	6 (1.5%)
**Precipitous labour**					
No	267 (95.4%)	67 (98.5%)	44 (100%)	17 (100%)	395 (96.6%)
Yes	13 (4.6%)	1 (1.5%)	0 (0%)	0 (0%)	14 (3.4%)
**Hyperstimulation**					
No	256 (91.4%)	68 (100%)	44 (100%)	17 (100%)	385 (94.1%)
Yes	24 (8.6%)	0 (0%)	0 (0%)	0 (0%)	24 (5.9%)
**Abruption**					
No	276 (98.6%)	66 (97.1%)	44 (100%)	17 (100%)	403 (98.5%)
Yes	4 (1.4%)	2 (2.9%)	0 (0%)	0 (0%)	6 (1.5%)
**Ruptured uterus**					
No	279 (99.6%)	67 (98.5%)	42 (95.5%)	17 (100%)	405 (99.0%)
Yes	1 (0.4%)	1 (1.5%)	2 (4.5%)	0 (0%)	4 (1.0%)
**Vasa previa**					
No	279 (99.6%)	67 (98.5%)	43 (97.7%)	16 (94.1%)	405 (99.0%)
Yes	1 (0.4%)	1 (1.5%)	1 (2.3%)	1 (5.9%)	4 (1.0%)
**Other intrapartum hemorrhage**					
No	278 (99.3%)	66 (97.1%)	43 (97.7%)	17 (100%)	404 (98.8%)
Yes	2 (0.7%)	2 (2.9%)	1 (2.3%)	0 (0%)	5 (1.2%)
**Cord accident**					
No	249 (88.9%)	61 (89.7%)	40 (90.9%)	17 (100%)	367 (89.7%)
Yes	31 (11.1%)	7 (10.3%)	4 (9.1%)	0 (0%)	42 (10.3%)
**Prolapsed cord**					
No	275 (98.2%)	67 (98.5%)	44 (100%)	15 (88.2%)	401 (98.0%)
Yes	5 (1.8%)	1 (1.5%)	0 (0%)	2 (11.8%)	8 (2.0%)
**Shoulder dystocia**					
No	268 (95.7%)	60 (88.2%)	37 (84.1%)	16 (94.1%)	381 (93.2%)
Yes	12 (4.3%)	8 (11.8%)	7 (15.9%)	1 (5.9%)	28 (6.8%)
**Other intrapartum complications**					
No	205 (73.2%)	36 (52.9%)	26 (59.1%)	9 (52.9%)	276 (67.5%)
Yes	75 (26.8%)	32 (47.1%)	18 (40.9%)	8 (47.1%)	133 (32.5%)
**Intrapartum complications total**					
Mean (SD)	0.600 (0.736)	0.809 (0.697)	0.750 (0.534)	0.706 (0.470)	0.655 (0.704)

	PA (n=280)	Mild (n=68)	Moderate (n=44)	Severe (n=17)	Overall (n=409)
Median [Min, Max]	0.00 [0.00, 3.00]	1.00 [0.00, 3.00]	1.00 [0.00, 2.00]	1.00 [0.00, 1.00]	1.00 [0.00, 3.00]
**Liquor colour**					
Clear	139 (49.6%)	32 (47.1%)	24 (54.5%)	4 (23.5%)	199 (48.7%)
Blood stained	16 (5.7%)	6 (8.8%)	1 (2.3%)	3 (17.6%)	26 (6.4%)
Meconium I	27 (9.6%)	2 (2.9%)	4 (9.1%)	1 (5.9%)	34 (8.3%)
Meconium II	31 (11.1%)	3 (4.4%)	5 (11.4%)	3 (17.6%)	42 (10.3%)
Meconium III	22 (7.9%)	5 (7.4%)	4 (9.1%)	1 (5.9%)	32 (7.8%)
Missing	45 (16.1%)	20 (29.4%)	6 (13.6%)	5 (29.4%)	76 (18.6%)
**1-min Apgar**					
Mean (SD)	6.06 (2.37)	2.96 (1.96)	2.60 (2.23)	1.12 (1.36)	4.98 (2.79)
Median [Min, Max]	6.00 [0.00, 9.00]	3.00 [0.00, 9.00]	2.00 [0.00, 9.00]	1.00 [0.00, 5.00]	5.00 [0.00, 9.00]
Missing	0 (0%)	0 (0%)	2 (4.5%)	0 (0%)	2 (0.5%)
**5-min Apgar**					
Mean (SD)	8.28 (1.75)	5.26 (2.03)	3.88 (2.24)	2.53 (2.29)	7.08 (2.64)
Median [Min, Max]	9.00 [2.00, 10.0]	5.00 [1.00, 9.00]	4.00 [0.00, 10.0]	2.00 [0.00, 7.00]	8.00 [0.00, 10.0]
Missing	0 (0%)	0 (0%)	1 (2.3%)	0 (0%)	1 (0.2%)
**10-min Apgar**					
Mean (SD)	9.39 (1.20)	7.39 (1.65)	5.54 (2.35)	3.88 (2.28)	8.42 (2.20)
Median [Min, Max]	10.0 [2.00, 10.0]	7.00 [4.00, 10.0]	5.00 [0.00, 10.0]	4.00 [0.00, 8.00]	10.0 [0.00, 10.0]
Missing	21 (7.5%)	9 (13.2%)	3 (6.8%)	1 (5.9%)	34 (8.3%)
**Lowest cord pH**					
Mean (SD)	7.04 (0.0877)	7.04 (0.149)	7.07 (0.227)	6.95 (0.168)	7.04 (0.125)
Median [Min, Max]	7.05 [6.77, 7.36]	7.02 [6.81, 7.40]	7.11 [6.26, 7.39]	6.99 [6.62, 7.19]	7.05 [6.26, 7.40]
Missing	5 (1.8%)	11 (16.2%)	2 (4.5%)	2 (11.8%)	20 (4.9%)
**Umbilical cord pH**					
Mean (SD)	7.20 (0.142)	7.06 (0.145)	7.04 (0.133)	6.86 (0.213)	7.11 (0.173)
Median [Min, Max]	7.22 [6.76, 7.46]	7.04 [6.76, 7.39]	7.02 [6.78, 7.30]	6.80 [6.56, 7.34]	7.13 [6.56, 7.46]
Missing	165 (58.9%)	4 (5.9%)	3 (6.8%)	4 (23.5%)	176 (43.0%)
**Initial base defecit**					
Mean (SD)	6.87 (4.48)	12.2 (4.31)	14.8 (5.00)	20.9 (6.97)	10.5 (6.17)
Median [Min, Max]	6.10 [0.100, 27.0]	12.7 [3.10, 23.1]	14.7 [2.30, 24.0]	22.7 [5.80, 30.0]	9.90 [0.100, 30.0]
Missing	175 (62.5%)	5 (7.4%)	7 (15.9%)	5 (29.4%)	192 (46.9%)
**Initial lactate**					
Mean (SD)	6.25 (2.91)	9.42 (3.00)	11.2 (3.11)	15.1 (5.81)	8.42 (4.04)
Median [Min, Max]	6.00 [1.10, 17.2]	9.70 [1.80, 16.9]	11.5 [4.70, 18.2]	16.7 [3.50, 23.0]	8.20 [1.10, 23.0]
Missing	186 (66.4%)	9 (13.2%)	19 (43.2%)	6 (35.3%)	220 (53.8%)
**Source of pH**					
ABG	2 (0.7%)	4 (5.9%)	9 (20.5%)	5 (29.4%)	20 (4.9%)
VBG	55 (19.6%)	42 (61.8%)	17 (38.6%)	3 (17.6%)	117 (28.6%)
CBG	57 (20.4%)	17 (25.0%)	9 (20.5%)	5 (29.4%)	88 (21.5%)
Missing	166 (59.3%)	5 (7.4%)	9 (20.5%)	4 (23.5%)	184 (45.0%)
**Most intensive resuscitation**					
None	110 (39.3%)	2 (2.9%)	2 (4.5%)	0 (0%)	114 (27.9%)
Facial oxygen	9 (3.2%)	2 (2.9%)	0 (0%)	0 (0%)	11 (2.7%)
BMV/IPPV	113 (40.4%)	36 (52.9%)	15 (34.1%)	2 (11.8%)	166 (40.6%)
Intubation	8 (2.9%)	14 (20.6%)	10 (22.7%)	2 (11.8%)	34 (8.3%)
CPR	5 (1.8%)	11 (16.2%)	12 (27.3%)	3 (17.6%)	31 (7.6%)
CPR and adrenaline	1 (0.4%)	0 (0%)	4 (9.1%)	9 (52.9%)	14 (3.4%)
CPAP/PEEP	28 (10.0%)	3 (4.4%)	0 (0%)	1 (5.9%)	32 (7.8%)
Missing	6 (2.1%)	0 (0%)	1 (2.3%)	0 (0%)	7 (1.7%)
**Assisted ventilation at 10 mins**					
No	258 (92.1%)	50 (73.5%)	15 (34.1%)	4 (23.5%)	327 (80.0%)
Yes	4 (1.4%)	14 (20.6%)	28 (63.6%)	9 (52.9%)	55 (13.4%)
Missing	18 (6.4%)	4 (5.9%)	1 (2.3%)	4 (23.5%)	27 (6.6%)
**Head circumference**					
Mean (SD)	35.2 (1.45)	35.4 (1.13)	35.3 (1.74)	36.0 (1.98)	35.3 (1.45)
Median [Min, Max]	35.0 [31.7, 39.5]	35.4 [33.0, 38.0]	35.3 [29.5, 39.0]	35.6 [33.5, 40.0]	35.1 [29.5, 40.0]
Missing	65 (23.2%)	10 (14.7%)	6 (13.6%)	7 (41.2%)	88 (21.5%)
**Baby length**					
Mean (SD)	50.9 (2.05)	52.3 (1.47)	52.5 (2.93)	50.0 (1.63)	51.3 (2.22)
Median [Min, Max]	51.0 [47.0, 56.0]	52.0 [49.0, 56.0]	53.0 [44.0, 58.0]	50.0 [48.0, 52.0]	51.0 [44.0, 58.0]
Missing	153 (54.6%)	44 (64.7%)	18 (40.9%)	13 (76.5%)	228 (55.7%)
