# Supplementary material for: Predictive modelling of hypoxic ischaemic encephalopathy risk following perinatal asphyxia
Source: Heliyon. 2021 Jun 29;7(7):e07411. doi: 10.1016/j.heliyon.2021.e07411 (PMC8261660; doi:10.1016/j.heliyon.2021.e07411)
Supplement: Pages from HLY7411_source.pdf — Table showing the evaluation of Model 1 (no biochemical markers) on the independent test set. PA = perinatal asphyxia without encephalopathy, HIE = hypoxic ischaemic encephalopathy, Mild = mild HIE, Mod = moderate HIE, Severe = severe HIE, TP = true positive, FP = false positive, TN = true negative, FN = false negative. [file mmc2.pdf]

	PA Vs HIE (all grades)	PA Vs Mild/Mod	PA Vs Mod/Severe	PA/Mild Vs Mod/Severe
TP	30	24	13	10
FP	9	10	1	3
TN	71	70	79	101
FN	12	13	5	8

Table S2. Table showing the evaluation of Model 1 (no biochemical markers) on the independent test set. PA = perinatal asphyxia without encephalopathy, HIE = hypoxic ischaemic encephalopathy, Mild = mild HIE, Mod = moderate HIE, Severe = severe HIE, TP = true positive, FP = false positive, TN = true negative, FN = false negative.
