# Supplementary material for: Predictive modelling of hypoxic ischaemic encephalopathy risk following perinatal asphyxia
Source: Heliyon. 2021 Jun 29;7(7):e07411. doi: 10.1016/j.heliyon.2021.e07411 (PMC8261660; doi:10.1016/j.heliyon.2021.e07411)
Supplement: Pages from HLY7411_source.pdf — Table showing the evaluation of Model 2 (including biochemical markers) on the independent test set. PA = perinatal asphyxia without encephalopathy, HIE = hypoxic ischaemic encephalopathy, Mild = mild HIE, Mod = moderate HIE, Severe = severe HIE, TP = true positive, FP = false positive, TN = true negative, FN = false negative. [file mmc3.pdf]

	PA Vs HIE (all grades)	PA Vs Mild/Mod	PA Vs Mod/Severe	PA/Mild Vs Mod/Severe
TP	29	24	16	10
FP	8	7	3	4
TN	28	29	33	50
FN	5	5	0	6

Table S3. Table showing the evaluation of Model 2 (including biochemical markers) on the independent test set. PA = perinatal asphyxia without encephalopathy, HIE = hypoxic ischaemic encephalopathy, Mild = mild HIE, Mod = moderate HIE, Severe = severe HIE, TP = true positive, FP = false positive, TN = true negative, FN = false negative.

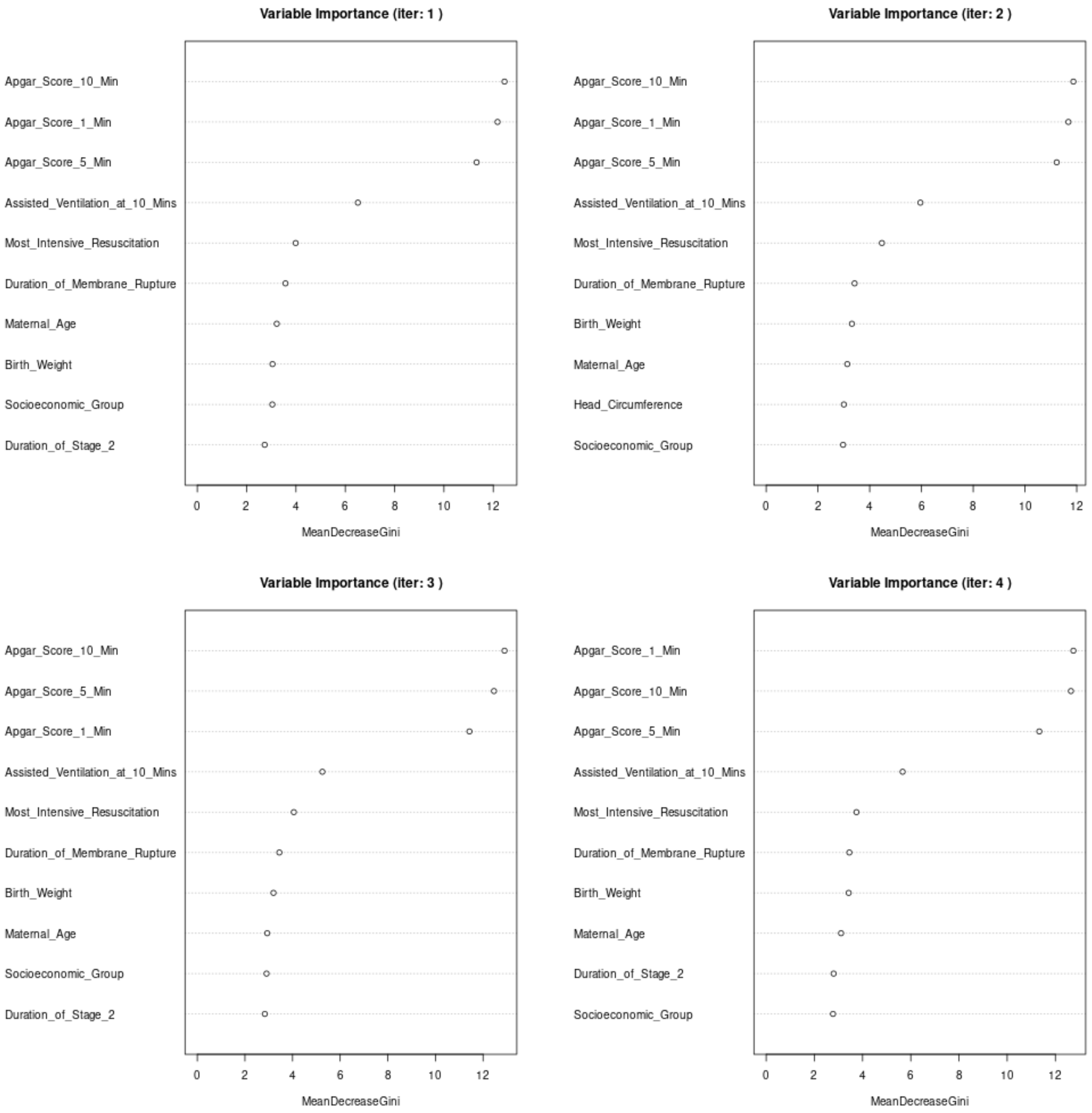

Figure S1. Variable importance plots for Model 1 (biochemical markers of pH, lactate and base deficit and lowest cord pH removed)

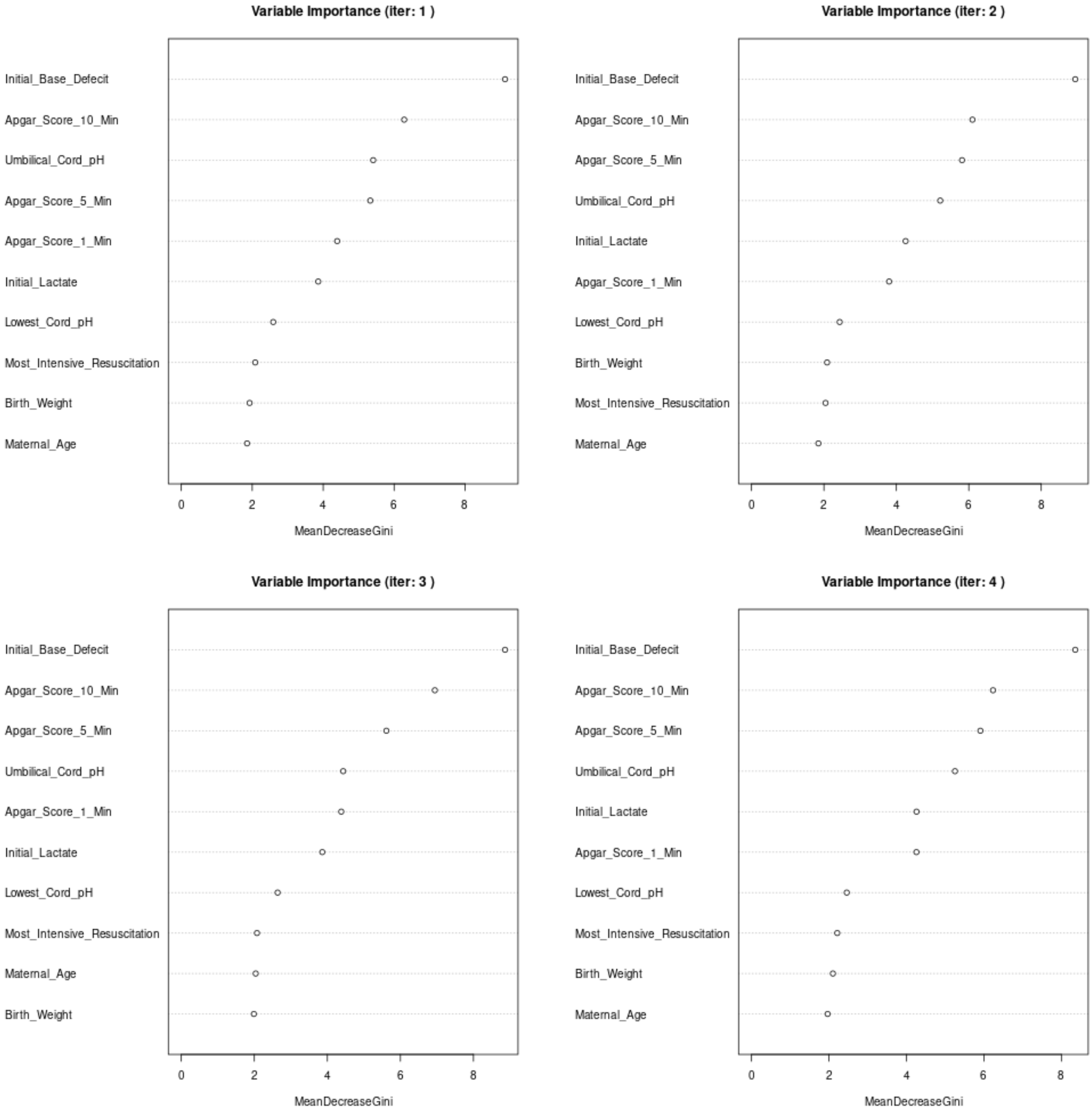

Figure S2. Variable importance plots for Model 2 (infants with missing pH, lactate and base deficit removed)
